# Assessment of cardiopulmonary resuscitation knowledge and skills among healthcare providers at an urban tertiary referral hospital in Tanzania

**DOI:** 10.1186/s12913-018-3725-2

**Published:** 2018-12-04

**Authors:** Winfrida T. Kaihula, Hendry R. Sawe, Michael S. Runyon, Brittany L. Murray

**Affiliations:** 1 Emergency Medicine Department, Muhimbili National Hospital, P.O. Box 65001, Dar es Salaam, Tanzania; 2 Emergency Medicine Department, Muhimbili University of Health and Allied Science, Dar es Salaam, Tanzania; 3 Department of Emergency Medicine, Carolinas Medical Center, Charlotte, NC USA; 4 Division of Paediatric Emergency Medicine, Emory University School of Medicine, Atlanta, GA USA

**Keywords:** Emergency department, Cardiopulmonary arrest, Cardiopulmonary resuscitation, Provider, Knowledge

## Abstract

**Background:**

Early and effective CPR increases both survival rate and post-arrest quality of life. In limited resource countries like Tanzania, there is scarce data describing the basic knowledge of CPR among Healthcare providers (HCP). This study aimed to determine the current level of knowledge on, and ability to perform, CPR among HCP at Muhimbili National Hospital (MNH).

**Methods:**

This was a descriptive cross sectional study of a random sample of 350 HCP from all cadres and departments at MNH from October 2015 to March 2016. Each participant completed a with 25 question multiple choice and fill-in-the-blank CPR test and a practical test using a CPR manikin where the participant was videotaped for 1–2 min. Two expert observers independently viewed the videos and rated participant performance on a structured data form. The primary outcome of interest was staff member overall performance on the written and practical CPR testing.

**Results:**

We enrolled 350 HCPs from all 12 MNH clinical departments. The median participant age was 35 (IQR 29–43) years, 225 (64%) were female and 138 (39%) had clinical experience of less than 5 years. Only 57 (16%) and 88 (25%) scored above 50% in written and practical tests, respectively according to local minimum passing test score and 13(4%) and 30 (9%) scored above 75% in written and practical tests, respectively according to international minimum passing test score on CPR. The 233(67%) HCP who reported prior experience performing CPR on an adult patient scored higher on testing than those without; 40% (IQR 28–54) versus 26% (IQR 16–42) respectively, but both groups had median scores <50%.

**Conclusion:**

The level of CPR knowledge and skills displayed by all cadres and in all departments was poor despite the fact that most providers reported having performed CPR in the past. Since MNH is a tertiary referral hospital, it may reflect the performance of resuscitation status of other local health centers in Tanzania and other low-income countries to employ a formal system of training every HCP in CPR. Staff should be certified and assessed regularly to ensure retention of resuscitation knowledge and skills.

**Electronic supplementary material:**

The online version of this article (10.1186/s12913-018-3725-2) contains supplementary material, which is available to authorized users.

## Background

Early recognition and intervention in cardiac arrest saves lives [1], For every minute without cardiopulmonary resuscitation (CPR) and defibrillation, the victim’s chance of survival from cardiac arrest decreases by 7–10% [2]. CPR is now modified into a simple version of skills that can be learned by anyone regardless of formal medical training [3]. In the hospital, this allows any trained staff member to rapidly initiate this live saving treatment [4].

In the developed world, the incidence of in-hospital cardiac arrest attended by a resuscitation teams and receiving CPR is approximately 2 per 1000 admissions [5]. In such settings, the training of staff members and the introduction of resuscitation teams and pre-employment requirements of CPR certification have resulted in improvement of outcomes of in-hospital cardiac arrest [6]. In developing countries there is limited published data on the incidence, organization and outcomes of in-hospital CPR [7]. Furthermore, in most parts of Africa, studies have shown health care providers (HCPs) to have poor knowledge and skills of CPR despite attempting to do resuscitation [8–10].

Since CPR is a very important skill, HCPs, irrespective of their training level or work setting should be competent in initiating and performing CPR, and hospitals should provide training to their staff [11, 12]. The knowledge of CPR amongst HCPs is significantly influenced by training [13] and it is a major determinant of the success of CPR [14]. To acquire this knowledge, routine training on CPR should be emphasized [15]. International recommendations dictate that HCPs repeat a CPR course at least every 2 years [14–16]. In many developing countries, standard resuscitation training is not routine [14, 17]. In fact, it is often simply assumed that all HCPs have the ability to recognize and treat cardiopulmonary arrest [18]. In Tanzania, the current knowledge and ability to perform CPR among HCPs is unknown.

This study aimed to determine the CPR knowledge, skills, and training among HCPs at **Muhimbili National Hospital** (MNH). The hope is that an improved understanding of current state of CPR knowledge and skill among these providers will help guide future training and the implementation of strategies that will benefit healthcare delivery to patients with cardiopulmonary arrest in low resource in-hospital settings.

## Methods

### Study design

This was a descriptive cross sectional study conducted at Muhimbili National Hospital (MNH) in Dar es Salaam, Tanzania from October 2015 to March 2016. MNH is the national referral hospital.

### Study setting and population

MNH is a National referral hospital with 1500-beds that receives patients from all over Tanzania. The Hospital had annual volume of 446,000 outpatients, 60,000 emergency department patients, and 53,000 admissions to general wards. The annual theater use is around 13,000 patients, while approximately 220 patients are admitted to Intensive Care Units annually [19]. At the time of the study, MNH had 2326 HCPs employed in the in-patient setting at the time of this study.

A sample of 350 staff representing 15% all staff providing inpatient care (which also represents 15% of each cadre in their respective departments) were randomly invited and consented to participate in the study during 6-month period of data collection. Cadres included health attendants, nurses, interns, registrars (general practitioners), residents (doctors in specialty training), specialists, and supporting clinical staff. Departments included the 11 major departments of MNH, and the intern doctors as a separate ‘department’ because they have their own classification within the hospital management scheme.

### Study protocol

Each participant completed a questionnaire that detailed basic demographics, clinical experience, and level of training, work site, Basic Life Support (BLS) /CPR certification and periodicity of BLS/CPR courses. Each participant then completed a 25 question multiple choice and fill-in-the-blank test (Additional file 1) which is derived from the American Heart Association (AHA) BLS test and modified to reflect local terminology and resources [1]. Each participant also completed a practical test using CPR manikins where the participant was videotaped for 1–2 min (Additional file 2). Two expert observers independently viewed the videos and used a structured data form, based on AHA practical skills testing and modified to local resource availability, to rate participant performance of practical skills, including the initial approach and compression and ventilation techniques (Additional file 3). Both the multiple-choice test and video observation checklist were piloted on a small group of registrars from the ED who were not participants in the study for feedback before implementation. The individual scores from the multiple-choice test and video observation were assessed independently and also combined to produce an overall score.

### Key outcome measures

The primary outcome of interest was staff member performance on written and practical CPR testing. Secondary outcomes included the relation of the performance on testing to the participant work sites; self reported knowledge on CPR, clinical experience and formal CPR certification and training status.

### Data analysis

Study data were entered in to an Excel database (Microsoft Corporation, Redmond, Washington, USA) and analyzed with StatsDirect version 3.0.133 (StatsDirect Ltd., Cheshire, UK). Descriptive statistics, including counts (percentages) and medians (interquartile ranges [IQR]) are reported as appropriate. Comparisons among groups were performed using the Chi Square or Fisher’s exact test for proportions and the Kruskal-Wallis test, with post hoc pairwise testing according to the Dwass-Steel-Critchlow-Fligner procedure as indicated, for continuous variables. Two-sided *p*-values < 0.05 were considered significant. Inter-observer agreement for scoring of the video performances was quantified with the Cohen’s Kappa.

CPR knowledge scores were divided into three categories: The Muhimbili University and Allied Sciences (MUHAS) minimum passing criteria of 50% [20], the UK Resuscitation Council minimal passing score of 75% [21], and the AHA minimal passing score of 84%. These comparisons were made to compare the local performance to local and international standards for CPR competency of HCP.

## Results

### Demographics

A total of 350 participants from 12 MNH departments were selected and all responded by answering self-administered questionnaires and demonstrating practical aspects of CPR on a manikin. The median participant age was 35 (IQR 29–43) years, 225 (64%) were female and 138 (39%) had clinical experience of less than 5 years (Table 1). Inter-observer agreement for rating of participant performance by video review and completion of the structured scoring checklist revealed and observed agreement of 87% and a corresponding Cohen’s Kappa of 0.69.

**Table 1:** Demographics of study participants

Age in years (median)	35 (IQR 29–43)
Gender	*N* = 350
Female	225 (64%)
Cadre	*N* = 350
Specialist	39 (11%)
Resident	37 (11%)
Registrar	15 (4.2%)
Intern	19 (5.4%)
Nurses	158 (45%)
Health attendant	68 (19.4%)
Supporting clinical staff	14 (4%)
Department	*N* = 350
Anesthesia/ICU/OT	32 (9.1%)
Cardiovascular	24 (7%)
Diagnostic and laboratory	19 (5.4%)
Emergency medicine	25 (7%)
General surgery	41 (12%)
Intern	19 (5.4%)
Internal medicine	39 (11.1%)
Obstetrics/Gynecology	51 (15%)
Pediatrics	40 (11%)
Psychiatry	19 (5.4%)
Radiology	19 (5.4%)
Surgical specialties	22 (6.2%)
Years of clinical practice	*N* = 350
Less than 5 years	156 (44%)
5 to 10 years	104 (30%)
More than 10 years	90 (26%)

### Reported CPR experience and training

A total of 233 (67%) participants reported to have previously performed CPR on an adult patient; of these, 108 (46%) reported to have done CPR 1–3 times in the past 1 month. There was a significant difference in the overall performance on testing between those who had and had not performed CPR in the past with median test scores of 40%(IQR 28–54) and 26%(IQR 16–42) respectively, *p* = 0.001 (Table 2). Overall, 239 (68%) of participants reported to have prior knowledge on CPR and 155 (44%) reported to have had prior formal adult BLS/CPR training. There was a significant difference in testing scores between those with and without prior training with median test scores of 46% (IQR 36–58) and 26% (IQR 18–40) respectively, *p* = 0.001 (Table 2). With regards to time in clinical practice, the overall performance on testing was better among those with less than 10 years clinical experience compared to those with experience more than 10 years (*p* < 0.0001) (Table 2).

**Table 2:** Baseline CPR training, CPR experience, and test scores

	Number of participants	Total test score median (IQR) %	Difference between groups
Performance of CPR	*N* = 350		*P* = 0.001*
Performed CPR in lifetime	233 (67%)	40 (IQR 28–54)	
Has never performed CPR	117 (33%)	26 (IQR 16–42)	
Performance in the past 1 month	*N* = 233		*P* = 0.34** T = 3.33
- None	98 (42%)	37 (IQR 27–52)	
- 1–3 times	108 (46%)	42 (IQR 30–56)	
- 4–6 times	21 (9%)	46 (IQR 34–61)	
->6 times	6 (3%)	41 (IQR 37–60)	
Reported knowledge on CPR	*N* = 350		*P* = 0.001*
Yes	239 (68%)	40 (IQR 28–54)	
No	111 (32%)	26 (IQR 18–37)	
Prior formal BLS training	*N* = 350		*P* = 0.001*
Yes	155 (44%)	46 (IQR 36–58)	
No	195 (56%)	26 (IQR 18–40)	
How long ago?	*N* = 155		*P* = 0.29** T = 2.41
Less than 1 year	58 (37.4%)	48 (IQR 37–58)	
Less than 5 years	58 (37.4%)	47 (IQR 38–60)	
More than 5 years	39 (25.2%)	44 (IQR 34–53)	
Years of clinical practice	*N* = 350		*P* < 0.0001** T = 35.43
Less than 5 years	156 (44%)	42 (IQR 28–52)	
5 to 10 years	104 (30%)	40 (IQR 28–54)	
More than 10 years	90 (26%)	26 (IQR 18–36)^a^	

*Mann-Whitney test

**Kruskal-Wallis test

^a^Post hoc analysis shows that there is no significant difference between the Less than 5 year group and the 5 to 10 year group, but that the More than 10 year group scored significantly lower

#### Initial approach

**Fig. 1:**
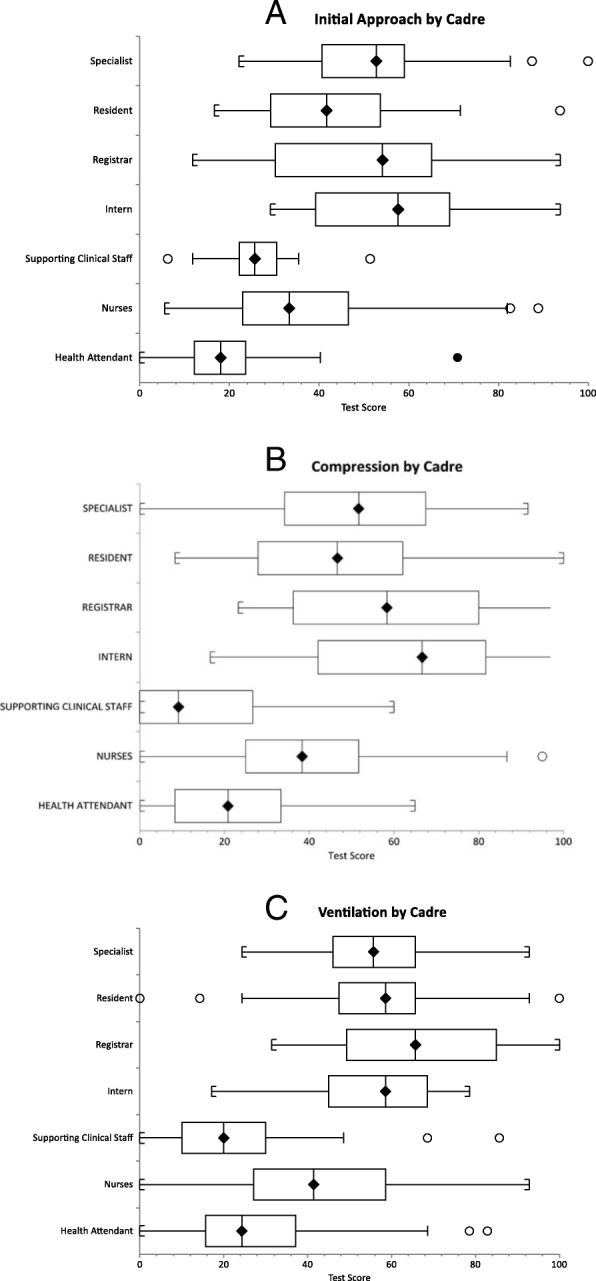
**a** Combined written and practical test scores of participants among different cadres on initial approach of cardiac arrest. **b** Combined written and practical test scores of participants among different cadres on principals of chest compression to the victim of cardiac arrest. **c** Combined written and practical test scores of participants among different cadres on principals of ventilation to the victim of cardiac arrest

The combined total score on initial approach (written and practical) among doctors was significantly higher than other cadres with no significance difference between the subgroups of doctors. Intern doctors scored higher, while health attendants followed by supporting clinical staff scored lower than other cadres (Fig. 1a).

#### Compressions

The combined total score on compressions (written and practical) among doctors was significantly higher than other cadres with no significance difference between their subgroups. Intern doctors and registrars scored higher, while supporting clinical staff followed by health attendants scored lower than other cadres (Fig. 1b).

#### Ventilation

The combined total score on ventilation components (written and practical) among doctors was significantly higher than other cadres with no significance difference between their subgroups. Registrars scored higher, while supporting clinical staff and health attendants scored lower than other cadres (Fig. 1c).

### Knowledge by department

#### Initial approach

**Fig. 2:**
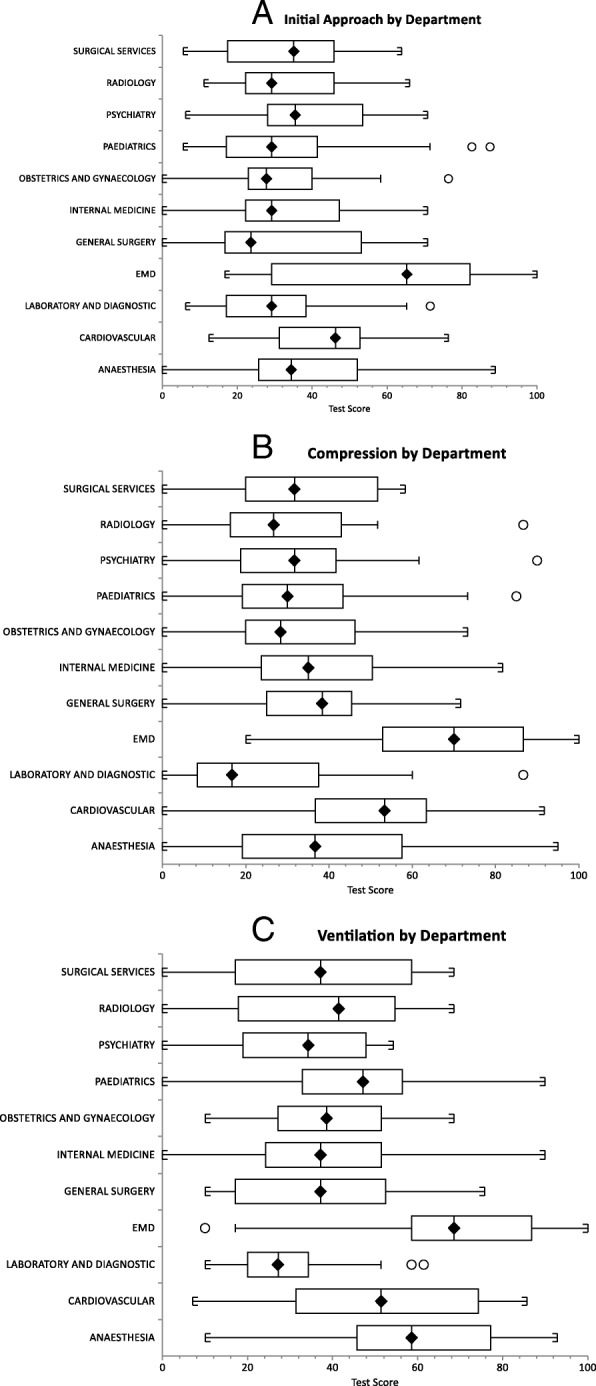
**a** Combined written and practical test scores of participants among different departments on initial approach of cardiac arrest. **b** Combined written and practical test scores of participants among different departments on principals of chest compression to the victim of cardiac arrest. **c** Combined written and practical test scores of participants among different departments on principals of ventilation to the victim of cardiac arrest

The combined total score on initial approach components (written and practical) was significantly higher for the ED than other departments, with no significance difference between the scores of the departments when compared with each other (Fig. 2a).

#### Compression

The combined total score on compressions components (written and practical) among doctors was significant higher for the ED than other departments, with no significance difference between the other departments when compared with each other (Fig. 2b).

#### Ventilation

The combined total score on ventilation components (written and practical) among doctors was significantly higher for the ED and anaesthesia than other departments, with no significance difference between the other departments when compared with each other (Fig. 2c).

### Overall knowledge among HCP

For the overall scores, 280 (80%) and 232 (66%) participants scored <50% in written and practical testing respectively. Only 13 (4%) and 30 (9%) participants scored >75% in written and practical testing respectively. Individual scores for components pertaining to initial approach, chest compression and ventilation are shown in Table 3.

**Table 3:** Test scores of all healthcare providers

Test component	Score < 50% *N* (%)	Score 50–74% *N* (%)	Score ≥ 75% *N* (%)
Initial approach (*N* = 350)			
Written test	223 (64%)	90 (26%)	37 (10%)
Practical test	244 (70%)	67 (19%)	39 (11%)
Compressions (*N* = 350)			
Written test	268 (77%)	57 (16%)	25 (7%)
Practical test	137 (39%)	140 (40%)	73 (21%)
Ventilation (*N* = 350)			
Written test	182 (52%)	95 (27%)	73 (21%)
Practical test	215 (61%)	105 (30%)	30 (9%)
Total scores (*N* = 350)			
Written test	280 (80%)	57 (16%)	13 (4%)
Practical test	232 (66%)	88 (25%)	30 (9%)

### Defibrillator use

A total of 197 (56%) participants reported familiarity with defibrillators, but no prior training in their use. Among these, 73 (37%) knew that defibrillation increase survival and only 6 (3%) asked for a defibrillator in practical skills testing. Of the 69 (20%) participants reported to have received prior training on defibrillator use, 25 (36%) knew that defibrillation can increase survival, and 3 (4%) asked for a defibrillator in practical skills testing. Of the 85 (24%) of all participants who reported having a defibrillator in their respective departments, only 4 (5%) asked for it in practical skills testing.

## Discussion

To the best of our knowledge this is the first study of knowledge on, and ability to perform CPR amongst HCPs of different cadres in Sub-Saharan Africa. Of the participants, 44% had clinical experience of less than 5 years. As HCP should be taught resuscitation skills in school, it may be expected of them to perform better. However this was not shown to be the case as they performed poorly on the tests, this may be due to not being trained in resuscitation skills during school or from loss of skills due not practicing them. It was also shown in this study that there was no association between duration of clinical experience and performance of CPR as there was no significance difference between participants who worked for less than 5 years and those between 5 and 10 years, which is a similar finding as in other studies [11, 22]. This suggests that there is not adequate basic life support training in school curricula. This also suggests that there is absence of continual in-service training that might have helped in improving HCP knowledge as seen in other studies [23, 24].

Though participants reported to have prior knowledge on CPR performance, their performance was poor overall. However, despite their poor performance, they still had significantly higher test scores than those who reported not to have knowledge of CPR. Participants who reported to have performed CPR infrequently have demonstrated poor performance. Infrequent performance of CPR has been shown to be associated with poor ability to retain CPR skills, and this may account for this finding [11]. Among participants, less than 50 % self-reported to have prior formal adult BLS/CPR training, with less than 40 % reporting to have the training in the last year. Though the performance is overall poor, this group scored higher than those without prior training. Again, previous studies have demonstrated an association between poor performance and lack of formal CPR training [23, 24]. This suggests that we must emphasize resuscitation programs and continual skill renewal programs in order to improve resuscitation skills [25].

The health attendants performed poorly on both tests and this may be explained by their limited role in patient care. Typically health attendants transport patients and assist nurses; and are often asked to help in emergency situations hence training in this group needs to be emphasized. Nurses likewise performed poorly on both the written and practical test. This poor performance is alarming as studies have shown nurses to often be the first responders to emergency situations in the wards [17, 22] and so training should also be emphasized among this cadre. Doctors in general performed poorly with a total score of 50%. Surprisingly, intern doctors performed better than specialist doctors on the written test and registrars performed better than specialist on the practical test. As specialists are senior and 64% reported to have received formal training on BLS/CPR, it was anticipated of them to perform best of all HCP. Interns on the other hand might have performed better since they have rotated in the ED where they have been exposed to CPR training and trained and practice.

Between the departments, the ED has been shown to perform better on combined score of the test. This may be explained by their regular involvement in resuscitation and training as compared to other departments. In fact, regular training and involvement has been found to improve CPR knowledge and performance in other settings, such as work by *Parajulee* et al. who showed that among nurses there was and association between worksite and total scores of participants due to training and experience [22].

As the local premier University of health in Tanzania the standard for passing is above 50% [24], 80% of HCP would have failed on the written test and 66% would have failed on the practical test as they scored less than 50%. However if we use international standard for passing score as a reflection of competency in BLS/CPR practice example the UK resuscitation council where the minimal passing score is ≥75 [25] and for AHA ≥84% [20], less than 10% of HCP would have passed the test and been considered competent.

Appropriate approach to cardiac arrest is known to impact the overall outcome of patients with cardiac arrest [26]. In our study, all HCP performed poorly on the written and practical assessment on the initial approach, with less than one third of HCP correctly identifying the importance of scene safety, checking for responsiveness and calling for help. These observations are similar to what has been reported in other low-and-middle income countries [27, 28], and contrary to findings of similar studies in high-income countries [29].

During CPR, correct technique of ventilation is key to as successes return of spontaneous circulation [30] interestingly the HCPs in our study population demonstrated a better score in written compared to other evaluated components of CPR. These findings of better theoretical knowledge may be a result of the fact that the old CPR guidelines prioritized teaching on airway (A) and breathing (B) above Circulation (C) in the approach to cardiac arrest assessment and management (the A-B-C approach) before the change to prioritizing Circulation above Airway and Breathing (the C-A-B approach). This observation was further supported by the performance in the practical assessment where nearly two-third of HCP delayed initiation of CPR while assessing and managing airway and breathing issues and performing prolonged pulse checks. The implication of this finding is that the MNH HCP does not seem to have received updated training on current CPR guidelines.

Though more than half of HCP reported to have knowledge of defibrillator use, only 37% knew that defibrillation increases chances of survival of a victim of cardiac arrest and less than 5 % actually called for it during practical aspect of CPR. This is an important knowledge gap as it has been shown that for every minute without CPR and defibrillation, the victim’s chance of survival from cardiac arrest decreases by 7 to 10% [2, 4]. Only 20% of HCP reported prior training in defibrillation and only 24% of HCP have a defibrillator in their departments. This lack of defibrillator access and training may contribute to suboptimal resuscitation efforts and increased mortality.

### Limitations

This is a single centered study done at the National Hospital, which may not be representative of other healthcare facilities in the region. Although the written test and practical skills testing checklist were not previously validated, the questionnaire and practical testing were designed based on published guidelines and were piloted locally prior to implementation. In fact, interobserver agreement was good between the expert observers who independently reviewed the videotapes and rated participant performance according to a standardized checklist.

It was said by the HCP that use of the English language was a barrier in the knowledge assessment, but the resulted demonstrated that there was not any improvement in performance on the practical test as compared to the written test.

## Conclusion

Before this study there was no information on the ability of HCP at MNH to recognize and treat cardiopulmonary arrest. Our data demonstrate overall poor knowledge, and performance, of CPR, for all HCP cadres and in all departments. Despite poor knowledge on CPR, HCP continue to provide resuscitative care and hence training should be mandated to improve their knowledge and performance in order to ensure optimal patient outcomes.

## Additional files

Additional file 1: Questionnaire. (DOC 111 kb)

Additional file 2: CPR assessment video. (M4V 22189 kb)

Additional file 3: Health care provider adult CPR/BLS skill demonstration checklist. (DOC 55 kb)

## Abbreviations

AED: Automated External Defibrillator
AHA: American Heart Association
BLS: Basic Life Support
CPR: Cardiopulmonary Resuscitation
ED: Emergency Department
EMS: Emergency Medical Services
ICU: Intensive Care Unit
ILCOR: International Liaison Committee on Resuscitation
MCQ: Multiple Choice Questions
ROSC: Return of Spontaneous Circulation

## References

[1] Field JM, Hazinski MF, Sayre MR, Chameides L, Schexnayder SM, Hemphill R (2010). Part 1: executive summary: 2010 American Heart Association guidelines for cardiopulmonary resuscitation and emergency cardiovascular care. Circulation.

[2] Cummins RO, Eisenberg MS, Hallstrom AP, Litwin PE (1985). Survival of out-of-hospital cardiac arrest with early initiation of cardiopulmonary resuscitation. Am J Emerg Med.

[3] Holmberg M, Holmberg S, Herlitz J, Swedish Cardiac Arrest Registry (2001). Factors modifying the effect of bystander cardiopulmonary resuscitation on survival in out-of-hospital cardiac arrest patients in Sweden. Eur Heart J.

[4] Smith GB (2010). In-hospital cardiac arrest: is it time for an in-hospital ‘chain of prevention’?. Resuscitation.

[5] Nolan JP, Soar J, Smith GB, Gwinnutt C, Parrott F, Power S (2014). Incidence and outcome of in-hospital cardiac arrest in the United Kingdom National Cardiac Arrest Audit. Resuscitation.

[6] Finn JC, Bhanji F, Lockey A, Monsieurs K, Frengley R, Iwami T (2015). Part 8: education, implementation, and teams: 2015 international consensus on cardiopulmonary resuscitation and emergency cardiovascular care science with treatment recommendations. Resuscitation.

[7] Wachira BW, Tyler MD (2015). Characterization of in-hospital cardiac arrest in adult patients at a tertiary hospital in Kenya. Afr J Emerg Med.

[8] Osinaike BB, Aderinto DA, Oyebamiji EO, Dairo MD, Diya KS. Evaluation of knowledge of doctors in a Nigrian tertiary hospital of CPR. Niger Med Pract [Internet]. 2008;52(1) Available from: http://www.ajol.info/index.php/nmp/article/view/28884. Cited 19 Feb 2018.

[9] Murila F, Obimbo MM, Musoke R. Assessment of knowledge on neonatal resuscitation amongst health care providers in Kenya. Pan Afr Med J [Internet]. 2012;11 Available from: https://www.ncbi.nlm.nih.gov/pmc/articles/PMC3361216/. Cited 19 Feb 2018. PMC3361216 22655112

[10] Silande O. Assessment of knowledge and skills of registered nurses regarding cardiopulmonary resuscitation at Muhimbili National Hospital, Dar es salaam Tanzania [Internet] [Thesis]: Muhimbili University of Health and Allied Sciences; 2010. Available from: http://ir.muhas.ac.tz:8080/jspui/handle/123456789/1058. Cited 19 Feb 2018

[11] Roshana S, KH B, RM P, MW S (2012). Basic life support: knowledge and attitude of medical/paramedical professionals. World J Emerg Med.

[12] Casey WF (1984). Cardiopulmonary resuscitation: a survey of standards among junior hospital doctors. J R Soc Med.

[13] Chamberlain DA, Hazinski MF, European Resuscitation Council, American Heart Association, Heart and Stroke Foundation of Canada, Resuscitation Council of Southern Africa (2003). Education in resuscitation: an ILCOR symposium: Utstein Abbey: Stavanger, Norway: June 22–24, 2001. Circulation.

[14] Govender K, Rangiah C, Ross A, Campbell L (2010). Retention of knowledge of and skills in cardiopulmonary resuscitation among healthcare providers after training. S Afr Fam Pract.

[15] Timerman S, Gonzalez MMC, Mesquita ET, Marques FRB, Ramires JAF, Quilici AP (2006). The international liaison committee on resuscitation (ILCOR): roll in guidelines 2005-2010 for cardiopulmonary resuscitation and emergency cardiovascular care. Arq Bras Cardiol.

[16] Cardiopulmonary Resuscitation: Statement by the ad hoc committee on cardiopulmonary resuscitation of the division of medical sciences, National Academy of Sciences—National Research Council. JAMA. 1966;198(4):372–9.

[17] Shrestha R (2011). Comparative study on level of knowledge of nursing personnel working in critical/general ward regarding cardio-pulmonary resuscitation (CPR) in BPKIHS, Dharan, Nepal. Australas Emerg Nurs J.

[18] Chandrasekaran S, Kumar S, Bhat SA, Saravanakumar, Shabbir PM, Chandrasekaran V (2010). Awareness of basic life support among medical, dental, nursing students and doctors. Indian J Anaesth.

[19] Home - Muhimbili National Hospital [Internet]. Available from: http://www.mnh.or.tz/. Cited 10 Mar 2013.

[20] MUHAS. Muhimbili University of Health and Allied Sciences [Internet]. MUHAS. 2018. https://www.muhas.ac.tz. [cited 26 Nov 2018].

[21] Soar J, Monsieurs KG, Ballance JHW, Barelli A, Biarent D, Greif R (2010). European resuscitation council guidelines for resuscitation 2010 section 9. Principles of education in resuscitation. Resuscitation.

[22] Parajulee S, Selvaraj V (2011). Knowledge of nurses towards cardiopulmonary resuscitation in a tertiary care teaching hospital in Nepal. J Clin Diagn Res.

[23] Broomberg J (1994). Managing the health care market in developing countries: prospects and problems. Health Policy Plan.

[24] Verplancke T, De Paepe P, Calle PA, De Regge M, Van Maele G, Monsieurs KG (2008). Determinants of the quality of basic life support by hospital nurses. Resuscitation.

[25] Narayan DPR, Biradar SV, Reddy MT, BK S (2015). Assessment of knowledge and attitude about basic life support among dental interns and postgraduate students in Bangalore city, India. World J Emerg Med.

[26] Truong HT, Low LS, Kern KB (2015). Current approaches to cardiopulmonary resuscitation. Curr Probl Cardiol.

[27] Rajeswaran L, Cox M, Moeng S, Tsima BM. Assessment of nurses’ cardiopulmonary resuscitation knowledge and skills within three district hospitals in Botswana. Afr J Prim Health Care Fam Med [Internet]. 2018;10(1) Available from: https://www.ncbi.nlm.nih.gov/pmc/articles/PMC5913783/. Cited 30 Jul 2018. 10.4102/phcfm.v10i1.1633 PMC5913783 29781687

[28] Alkandari SA, Alyahya L, Abdulwahab M (2017). Cardiopulmonary resuscitation knowledge and attitude among general dentists in Kuwait. World J Emerg Med.

[29] Malta Hansen C, Kragholm K, Pearson DA, Tyson C, Monk L, Myers B (2015). Association of bystander and first-responder intervention with survival after out-of-hospital cardiac arrest in North Carolina, 2010–2013. JAMA.

[30] Pan J, Zhu J-Y, Kee HS, Zhang Q, Lu Y-Q (2015). A review of compression, ventilation, defibrillation, drug treatment, and targeted temperature management in cardiopulmonary resuscitation. Chin Med J.

