# Supplementary material for: Assessment of cardiopulmonary resuscitation knowledge and skills among healthcare providers at an urban tertiary referral hospital in Tanzania
Source: BMC Health Serv Res. 2018 Dec 4;18:935. doi: 10.1186/s12913-018-3725-2 (PMC6278030; doi:10.1186/s12913-018-3725-2)
Supplement: Supplementary file 1 — Questionnaire. (DOC 111 kb) [file 12913_2018_3725_MOESM1_ESM.doc]

## **QUESTIONNAIRE** Q.NO _______

**Please tick in the boxes provided**

**DEMOGRAPHICS**

Age:

Gender: Male Female

**Level of training Work station**

Doctors: Super Specialist ________________

Specialist ­­­________________

Resident ________________

Registrar ________________

Intern doctor Period of rotation Supporting Clinical Staff ________________

Nurses: Nursing Officer ________________

Assistant Nursing Officer ________________

Enrolled nurse ________________

Health Attendant ________________

Years of clinical practice ________

Less than 5 years 5 to10 years More than 10 years

**WORK EXPERIENCE**

1. Have you ever tried to revive/resuscitate a dying person/adult with no pulse? Yes No
2. If yes how many times in the past one month
3. Do you know how to revive/resuscitate a dying person? Yes No
4. If no, do you think it is important to know as part of your job Yes No
5. Have you ever tried to revive/resuscitate a dying child with no pulse? Yes No

**If iii. Above is YES, please answer the questions below:**

1. Have you formally received training on Basic Life Support/CPR? Yes  No
2. How long ago?

Less than 1 year Less than 5 years More than 5 years

1. Do you know what a Defibrillator is? Yes No
2. Have you ever been trained to use a Defibrillator? Yes No
3. Do you have a Defibrillator in your department? Yes No

**WRITTEN TEST**

**Circle the best answer/fill in the blanks**

**If you don’t know the answer write IDN (I DON’T KNOW) besides the question.**

**If you can’t understand the language/terminology inform the researcher for Swahili interpretation verbally.**

1. What is the long form of “CPR” ______________________________________
2. You are alone and find a patient who is possibly in cardiac arrest. What is your first step?
   1. Check for responsiveness
   2. Ensure scene safety
   3. Begin Chest Compressions
   4. Open the airway and give 2 breaths
3. You are certain of the above answer. What is your next step?
   1. Begin chest compressions

|  |
| --- |

- 1. Check for responsiveness
  2. Call the patient’s doctor
  3. Provide rescue breathing

1. You are alone and find a patient who is in cardiac arrest, what should you do?
   1. Certify death
   2. Shout for help
   3. Take blood samples
   4. Check the blood pressure
2. What part of resuscitation of a patient with no pulse has been shown to improve survival?
   1. Early intubation
   2. Early defibrillation
   3. Giving adrenaline
   4. Placing a central line
3. The current American Heart Association guidelines for adult CPR recommend this order of steps:
   1. Chest compressions, Airway, Breathing
   2. Airway, Breathing, Check Pulse
   3. Airway, Breathing, Chest Compressions
   4. None of the above
4. When do you start CPR?
   1. There is no pulse and patient is not breathing
   2. There is a pulse and the patient is not breathing
   3. There is a pulse and the patient is unconscious
   4. All of above
5. Where should you attempt to perform a pulse check during CPR in adults?
   1. Brachial artery
   2. Ulnar artery
   3. Temporal artery
   4. Carotid artery
6. How long do you feel for a pulse during CPR?
   1. More than 10 minutes
   2. 1 hour
   3. Less than 10 seconds
   4. More than 10 seconds
7. Hand placement/position in CPR is:
   1. At the lower end of the chest and upper abdomen
   2. At the apex of the left part of the chest (On top of the heart)
   3. Centre of the breast bone between nipples
   4. Upper part of the breast bone above the nipples
8. The recommended rate for giving chest compressions in CPR is at least _______ compressions a minute.
9. The correct compression-ventilation ratio for adult CPR when done by one person is _______compressions to ______ breaths.
10. What is the recommended depth of chest compression in adults?
    1. At least 1 inches ( 2.54cm)
    2. At least 1½ (3.56cm)
    3. At least 2 inches (5cm)
    4. At least 4 inches (10cm)
11. Health care providers should try to minimize interruptions in chest compressions to less than _______ seconds.
12. The critical characteristic/s of high-quality CPR includes which of the following?
    1. Starting chest compressions within 10 seconds of recognition of cardiac arrest
    2. Push hard and fast
    3. Minimize interruptions
    4. All of the above
13. A simple technique for clearing a patient’s airway is:
    1. Head tilt, chin lift
    2. Push chin down, tilt head forward
    3. Lift chin up, turn head sideways
    4. Flex the neck
14. The following device is used when performing assisted ventilation during CPR in the hospital:
    1. A face shield
    2. Bag valve mask
    3. A face mask
    4. There is no preferred method
15. Correct ventilation during CPR is assured by:
    1. Visible abdominal rise
    2. Visible chest rise
    3. Coughing/gagging
    4. None of above
16. Rescue Breathing is used for a patient who is unconscious, but has a pulse. What is the correct rate for rescue breathing in an adult?
    1. 1 breath every 3 to 5 seconds
    2. 1 breath every 5 to 6 seconds
    3. 2 breaths every 3 to 5 seconds
    4. 2 breaths every 5 to 6 seconds
17. If you believe there is risk of a C-spine injury, the best way to open the airway is using
    1. Flexion technique
    2. Hyperextension technique
    3. Head tilt, chin lift technique
    4. Jaw thrust technique
18. How often should health care providers switch roles during CPR?
    1. After every cycle of CPR
    2. After every 2 cycles of CPR
    3. After every 5 cycles of CPR
    4. After every 10 cycles of CPR
19. The correct compression-ventilation ratio for children getting CPR when done by one person is _______compressions to ______ breaths.
20. What is the recommended depth of chest compressions in child aged 1 to puberty
    1. 1/3 to ½ of the depth of the chest
    2. At least ¼ of the depth of the chest
    3. 5cm or more
    4. 1cm or more
21. What is the correct rate for rescue breathing in a child?
    1. 1 breath every 3 to 5 seconds
    2. 1 breath every 5 to 6 seconds
    3. 2 breaths every 3 to 5 seconds
    4. 2 breaths every 5 to 6 seconds
22. For the best ventilation of infants, the head should be:
    1. Hyper extended
    2. Neutral position
    3. Flexed position
    4. Turned to left lateral position
