# Supplementary material for: Assessment of cardiopulmonary resuscitation knowledge and skills among healthcare providers at an urban tertiary referral hospital in Tanzania
Source: BMC Health Serv Res. 2018 Dec 4;18:935. doi: 10.1186/s12913-018-3725-2 (PMC6278030; doi:10.1186/s12913-018-3725-2)
Supplement: Supplementary file 3 — Health care provider adult CPR/BLS skill demonstration checklist. (DOC 55 kb) [file 12913_2018_3725_MOESM3_ESM.doc]

## **HEALTH CARE PROVIDER ADULT CPR/BLS SKILL DEMONSTRATION CHECKLIST**

Participant number ____________ Test date _____________________

During this phase we evaluate the health care provider’s ability to initiate BLS and deliver a high quality CPR

| Skill step | Critical criteria | Correct | Incorrect | Notes |
| --- | --- | --- | --- | --- |
| 1 | **CHECK FOR RESPONSE**   - Check for response (inflict pain, tapping, hello) |  |  |  |
|  | - Time should be within 10 seconds |  |  |  |
| 2 | **CALL FOR HELP**   - Call for help within 10 seconds |  |  |  |
|  | - Get defibrillator |  |  |  |
| 3 | **PULSE CHECK**   - Check for pulse not more than 10 seconds |  |  |  |
|  | - Carotid pulse |  |  |  |
|  | - Between two heads of sternocleidomastoid muscle |  |  |  |
|  | - Finger tips use to check pulse |  |  |  |
| 4 | **GIVE HIGH QUALITY CPR**   - Immediately not more than 10 seconds |  |  |  |
|  | - Correct position between the nipples, center of chest/lower half of the chest bone |  |  |  |
|  | - Two hands, one hand on top of the other using the heel of the first hand |  |  |  |
|  | - Extended elbow |  |  |  |
|  | - 30 compressions 10% lower and upper limit (record number) |  |  |  |
|  | - Adequate rate at least 100/min (count number of compression and divide with time on the video in the first cycle) |  |  |  |
|  | - Adequate depth at least 2inches in depth (75%) of compressions (There is a mark at the chin of the manikin) |  |  |  |
|  | Critical criteria | Correct | Incorrect | Notes |
|  | - Allows complete chest recoil (75% of compressions) |  |  |  |
|  | - Minimize interruptions, hands off not more than 10 seconds |  |  |  |
|  | - Hand should be in contact with the chest |  |  |  |
| 5 | **VENTILATION**   - Simple airway maneuvers (head tilt or chin lift) |  |  |  |
|  | - Correct technique of hand placement (fingers below the chin and above the bag) |  |  |  |
|  | - 2 breaths each over 1 second (within 10 seconds) |  |  |  |
|  | - Chest rise (All 2 breaths) |  |  |  |
|  | - Bag valve mask should cover the mouth and nose |  |  |  |
|  | - Compressions resumed immediately after breaths (within 10 seconds) |  |  |  |
|  | - From the end of the first compression to the start of the next compression time should be less than 10 seconds |  |  |  |
